# Web-based interactive mapping from data dictionaries to ontologies, with an application to cancer registry

**DOI:** 10.1186/s12911-020-01288-7

**Published:** 2020-12-15

**Authors:** Shiqiang Tao, Ningzhou Zeng, Isaac Hands, Joseph Hurt-Mueller, Eric B. Durbin, Licong Cui, Guo-Qiang Zhang

**Affiliations:** 1 The University of Texas Health Science Center at Houston, Houston, TX USA; 2 Department of Computer Science, University of Kentucky, Lexington, KY USA; 3 Kentucky Cancer Registry, Lexington, KY USA; 4 Department of Internal Medicine, University of Kentucky, Lexington, KY USA

**Keywords:** Data dictionary, Ontology, Concept mapping

## Abstract

**Background:**

The Kentucky Cancer Registry (KCR) is a central cancer registry for the state of Kentucky that receives data about incident cancer cases from all healthcare facilities in the state within 6 months of diagnosis. Similar to all other U.S. and Canadian cancer registries, KCR uses a data dictionary provided by the North American Association of Central Cancer Registries (NAACCR) for standardized data entry. The NAACCR data dictionary is not an ontological system. Mapping between the NAACCR data dictionary and the National Cancer Institute (NCI) Thesaurus (NCIt) will facilitate the enrichment, dissemination and utilization of cancer registry data. We introduce a web-based system, called Interactive Mapping Interface (IMI), for creating mappings from data dictionaries to ontologies, in particular from NAACCR to NCIt.

**Method:**

IMI has been designed as a general approach with three components: (1) ontology library; (2) mapping interface; and (3) recommendation engine. The ontology library provides a list of ontologies as targets for building mappings. The mapping interface consists of six modules: project management, mapping dashboard, access control, logs and comments, hierarchical visualization, and result review and export. The built-in recommendation engine automatically identifies a list of candidate concepts to facilitate the mapping process.

**Results:**

We report the architecture design and interface features of IMI. To validate our approach, we implemented an IMI prototype and pilot-tested features using the IMI interface to map a sample set of NAACCR data elements to NCIt concepts. 47 out of 301 NAACCR data elements have been mapped to NCIt concepts. Five branches of hierarchical tree have been identified from these mapped concepts for visual inspection.

**Conclusions:**

IMI provides an interactive, web-based interface for building mappings from data dictionaries to ontologies. Although our pilot-testing scope is limited, our results demonstrate feasibility using IMI for semantic enrichment of cancer registry data by mapping NAACCR data elements to NCIt concepts.

## Background

Ontologies have been commonly used to facilitate data management, data sharing, and information retrieval in biomedicine. To enhance semantic interoperability among ontologies, significant effort has been spent to study algorithms mapping concepts and relations between different ontologies [1]. Several ontology mapping systems and tools have been developed for this purpose [2–6] (an overview can be found at https://www.ontologymatching.org). However, few studies have focused on mappings between data dictionaries and ontologies, even though data dictionaries are essential for data collection. While mapping between ontologies takes place at a similar level of semantic abstraction, mapping between data dictionaries and ontologies takes place between different levels of semantic abstraction, and hence presents a more challenging situation. Moreover, mappings between data dictionaries and ontologies, when available, will benefit data-intensive biomedical applications where data are from different sources. For example, MEDCIS [7], X-search [8], and DataSphere [9] are semantically enabled cohort search applications that leverage ontologies and canonical data dictionaries. In the case of X-search, a canonical data dictionary is used to drive the interface for querying and harmonizing heterogeneous datasets in the National Sleep Research Resource [10–12]. To facilitate query translation, a set of mappings between the various dataset-specific data dictionaries and the canonical data dictionary have been manually created and maintained by a group of domain experts using spreadsheets. Such a file-based approach has limitations in terms of distributing the workload, facilitating collaborative review, and ensuring the quality of the mapping.

In this paper, we introduce a web-based Interactive Mapping Interface (IMI) for researchers to collaboratively build mappings between data dictionaries and ontologies. We report the architecture design and interface features of IMI. To validate our approach, we implemented an IMI prototype and pilot-tested features using the IMI interface to map a sample set of the North American Association of Central Cancer Registries (NAACCR) data elements to National Cancer Institute Thesaurus (NCIt) concepts. IMI has been successfully pilot-tested to construct a subset of mappings between the NAACCR data dictionary and NCIt.

## Methods

The overall architecture of IMI consists of three components: an ontology library, an interactive mapping interface, and a recommendation engine. The three components are integrated to support the general workflow of (1) importing the target ontology and source data dictionary; (2) performing mappings from the source data dictionary to the target ontology through the interactive interface; and (3) visualization and exporting of the mapping results.

### Ontology library

The IMI ontology library serves as the target source for mapping. It is managed and maintained by the application system administrator. Ontologies are imported in structured format (see BioPortal [13] for a rich source of biomedical ontologies) and can be populated into the backend NoSQL database such as MongoDB [14]. MongoDB was chosen as IMI’s backend database in order to provide flexibility in handling a large number of data elements, but any database should work [15]. The ontology library can be expanded using IMI’s management interface: the interface supports the importing of ontologies in the comma-separated values (CSV) format, with the data fields configurable. Ontologies often contain information beyond the scope of the mapping needs. Therefore, the ability to select fields to be imported is a desirable feature, making our ontology library more compact without sacrifice of intended roles.

### Interactive mapping interface

The interactive mapping interface of IMI consists of six seamlessly integrated modules: project management, mapping dashboard, access control, logs and comments, hierarchical visualization, and result review and exportation. The mapping interface provides an interactive process to support the mapping of one data element at a time. The access control module is implemented to manage users and grant or remove privileges. Logs and comments are used to track all mapping activities and enable information sharing during the mapping process. The module for logs and comments will be important for possible crowdsourcing of mapping tasks. Mapping results can be downloaded using the mapping export module. The ontological hierarchy visualization module renders the mapping results using the target ontology’s hierarchical structure as a reference background.

**Fig. 1:**
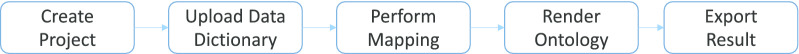
Five stems of IMI mapping workflow

The mapping workflow of IMI is demonstrated in Fig. 1 with five main steps: project creation, data dictionary upload, mapping data elements to ontological concepts, visualization of mapped concepts in the corresponding ontological hierarchy, and export mapping results as a file.

#### Project management

The mapping process begins with the creation of a new project, with the goal of mapping data elements in a data dictionary to concepts in an ontology (the target ontology). There are several required inputs for a new project. First, the project owner needs to select the target ontology from the ontology library. Second, the project owner needs to select from two choices, a public project or a private project (which is the default). If the project is public, its content can be accessed by all users in IMI. Otherwise, the project can only be accessible by users with permission granted by the project owner. Users assigned to a project can access the project from their own project management interface. The project owner can further configure which aspects of the target ontology will be displayed in the interface, so that aspects irrelevant to the mapping task will not be shown on the interface. After the creation and configuration of a project, selected users can proceed to the mapping interface to perform mapping tasks collaboratively and/or distributively.

#### Data dictionary upload

A source data dictionary needs to be uploaded in order to perform mappings. IMI makes the upload process easy by providing a data dictionary uploading interface. Users can specify the fields of variables (or data elements) to import, the fields to be displayed in the mapping interface, and the fields to show when a variable in the data dictionary is selected.

#### Mapping

The mapping interface consists of three main areas: (1) an area to list all the uploaded variables from the source data dictionary; (2) an area to show the details of the selected variable; and (3) an area to show the top (say five) recommended concepts in the target ontology and the details of the selected concept.

There are two modes for reviewing variables from the source data dictionary: browsing and search. The browsing mode provides a list view of all variables so that users can explore them one by one. The search mode enables expert users to directly search for variables by keywords. Along with the variable name, a color-coded visual indicator displays the mapping status of the variable along with the number of mapping comments entered for the variable. A green box with the character "M" indicates that the variable has been mapped while a red box with the character "U" indicates that the variable is unmapped.

When a variable from area 1 is selected from the source data dictionary, area 2 will show its details. The message icon on the top right of area 3 is used to open the logs and comments, where the user can view the mapping activities and comments from other users. When the selected variable is not mapped, a candidate list of recommended concepts from the target ontology will be fetched and showed in area 3. Below the candidate list, an additional search widget is provided for the user to search for other concepts in the target ontology. Once a matching concept is identified, the user may click the match button to complete a mapping. Once the variable is mapped, the list of recommendations is replaced with the details of the mapped concept.

#### Visualization and result export

After the mapping is completed, the target ontology’s hierarchical structure may be leveraged to visualize the hierarchical organization of the source data dictionary. For our IMI prototype, the visualization module is implemented with the Data-Driven Documents (D3) JavaScript library [16]. The hierarchical presentation of the ontology can be viewed as multiple trees (i.e., a forest). Each root concept or top-level concept corresponds to the root of a tree. To visualize the hierarchical structure, we treat each mapped concept as a leaf node and trace all the way back to the root node in the target ontology while gathering all child nodes to display as intermediate nodes. The results are represented using a nested array which are passed to the D3 environment for rendering.

The mapping results also can be exported in CSV format using the exportation module. With specific columns defined in the CSV file, IMI can import it back while preserving all completed mappings.

### Recommendation engine

IMI features an automated recommendation engine. When an unmapped data element is selected from the source data dictionary, a list of recommendation concepts from the target ontology is automatically generated and displayed. In our IMI prototype, this is accomplished using a fuzzy matching algorithm [17]. The fuzzy matching algorithm calculates the similarity between word sequences and returns a score to represent the similarity. IMI uses a priority queue to keep track of the top ten concepts from target ontology with the highest scores. The list of recommended concepts can be generated on-the-fly but the response time is dependent on the size of the target ontology.

### Evaluation method

We prototyped an IMI system and performed preliminary evaluation to assess the functional design of the IMI mapping interface and demonstrate the feasibility of our interfaces in terms of its ability to properly perform the functions of our design. We compare IMI mapping with file-based mapping to highlight IMI features. We assess time taken to perform mappings using both approaches. Although the performance of the recommendation engine is not a primary focus, we evaluate its usability by comparing top candidate(s) recommended with the mapping results obtained by domain experts. The percentages of correct mapping recommendations are reported.

Mappings have been performed between the NAACCR variables used in the Kentucky Cancer Registry (KCR) and the NCIt concepts using IMI. The KCR was established at the University of Kentucky Markey Cancer Center in 1991. KCR is a central cancer registry that receives data about incident cancer cases from all healthcare facilities and physicians in Kentucky within 6 months of diagnosis. KCR, like all other U.S. and Canadian cancer registries, utilizes the standardized data dictionary provided by the NAACCR to collect patient data [18]. To reduce the data access barriers and facilitate query and exploration of cancer registry data resources, we needed to build a faceted query system by reusing the NCIt ontology system [19], where a mapping between the NAACCR data dictionary and NCIt was required.

## Results

We implemented IMI using Ruby on Rails, an agile web development framework. IMI has been deployed and is publicly available at https://epi-tome.com. The mapping workflow is initiated by creating a project using our project management module. The project management module uses a standard CRUD (create, read, update, delete) interface where users can specify the project name, project description, and select the target ontology and one default search field. The default search field for the target ontology configures the search field (e.g., preferred label) when users try to search matching concepts from the target ontology. Users can also make their projects publicly available. All users of IMI are able to contribute to the mappings for public projects. Once a project is created, the workflow proceeds to the data dictionary uploading interface, which has a similar mechanism as the ontology uploader.

IMI supports ontology import from a CSV file with the same format as provided in BioPortal. To add a new ontology, a user with system administrator role simply click the “add a new ontology” button. When an ontology file in the CSV format is selected from the local disk, IMI scans and retrieves the header of the CSV file. Then the user is provided with an option to select which fields to import into IMI. For our prototype, IMI has imported NCIt with over 150,000 concepts. Additional ontologies can be incorporated into the ontology library as needed.

### Experiment

We performed an experiment to validate the IMI design features and functionalities. A total of 301 NAACCR variables were extracted from the KCR registry data and the extraction results were verified by domain experts from KCR (authors IH, JM, and EBD) with experience in both NAACCR and NCIt. Overall, 47 out of 301 variables were successfully mapped to NCIt concepts (Table 1). Five branches of hierarchical trees were constructed from NCIt.

**Table 1:** NAACCR variables that are mapped to NCIt concepts

Variable from NAACCR	Mapped concept from NCIt
Race 1	Race
Race 2	Race
Race 3	Race
Race 4	Race
Race 5	Race
Spanish/Hispanic Origin	Hispanic or Latino
Computed Ethnicity	Computed Ethnicity Code
Computed Ethnicity Source	Computed Ethnicity Source Code
Sex	Sex
Date Of Birth	Birth Date
Birthplace-State	Birth State Code
Birthplace-Country	Birth Country Code
Date Of Last Contact	Date of Last Contact
Vital Status	Vital Status
Addr Current-City	City
Addr Current-State	US State
Addr Current-Postal Code	Postal Code
Cause Of Death	Cause of Death
Autopsy	Autopsy Indicator
Patient System Id-Hosp	Patient Identifier
Marital Status At Dx	Marital Status Code at Diagnosis
Age At Diagnosis	Age at Diagnosis
Ruralurban Continuum 2003	Rural-Urban Continuum Code 2003
Census Tract 2010	Census Tract
Ruralurban Continuum 2013	Rural-Urban Continuum Codes 2013
Date Of Diagnosis	Initial Cancer Diagnosis Date
Primary Site	Primary Site of Disease
Laterality	Laterality
Histologic Type Icd-O-3	Histology Type Code ICD-O-3
Diagnostic Confirmation	Diagnostic Confirmation Code
Type Of Reporting Source	Reporting Source Type Code
Class Of Case	Class of Case
Primary Payer At Dx	Primary Healthcare Payer
Regional Nodes Positive	Number of Regional Lymph Nodes Positive
Regional Nodes Examined	Number of Regional Lymph Nodes Examined
Rx Summ-Surgical Margins	Surgical Margin
Vendor Name	Vendor Name
Follow-Up Source	Last Follow-up Source Type Code
Place Of Death	Location of Death
Text-Usual Occupation	Occupation
Tnm Clin T	AJCC v7-Primary Tumor (T)
Tumor Size Summary	Tumor Size Measurement
Derived Ajcc-6 Stage Grp	AJCC v6 Stage
Multiplicity Counter	Number of Primary Tumors in this Location
Lymph-Vascular Invasion	Is Lymphatic Invasion Present
Seer Summary Stage 2000	SEER Summary Stage 2000
Registry Id	Cancer Registry Identifier

### Mapping dashboard

Mapping dashboard is the core module for IMI. From the mapping dashboard, users can navigate to other modules including access control, logs and comments, visualization, and result review and exportation.

**Fig. 2:**
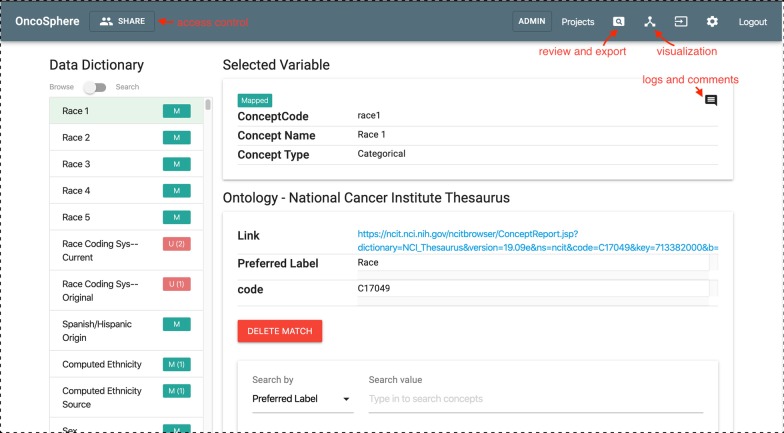
Mapping dashboard

Figure 2 shows the mapping dashboard with the four areas annotated. The “Data Dictionary” area lists all the variables in the uploaded data dictionary. The default mode is the browsing mode and users can switch to search mode using the switch widget. The “Selected Variable” area shows the variable selected from the “Data Dictionary” area. Below the “Selected Variable” area is the target ontology area. If the variable is already mapped, the mapped concept from target ontology will be shown in this area. Users can delete the existing match and utilize the search widget down below to search other candidates and perform mapping again. In this example, we can see the variable “Race 1” from the NAACCR data dictionary is mapped to the NCIt concept “Race.” If the concept is currently unmapped, a list of recommendations will be displayed and ranked by the similarity scores.

**Fig. 3:**
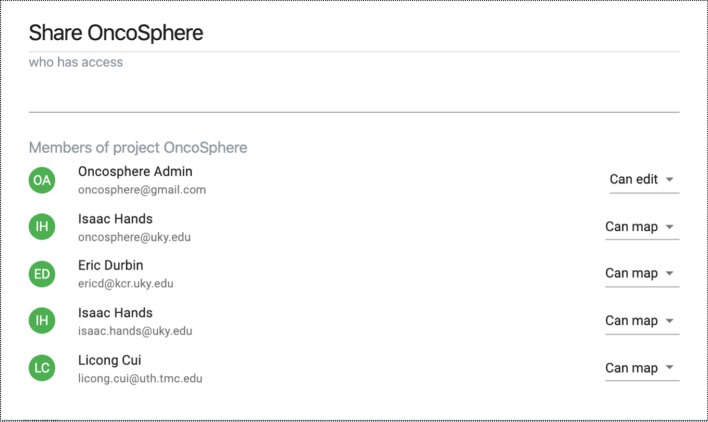
Access control module with uses on the left and privilege on the right

Figure 3 shows the access control module as well as the module for logs and comments. If the current project is not public, the project owner can use the access control module to grant privileges to certain users. The access control module provides two privileges: “can edit” and “can map.” The first privilege is the administrator level privilege while the second one only allows users to perform mappings. The module for logs and comments keeps track of each mapping and mapping-removing activities. Users are permitted to provide comments about current mappings.

### Mapping result visualization

**Fig. 4:**
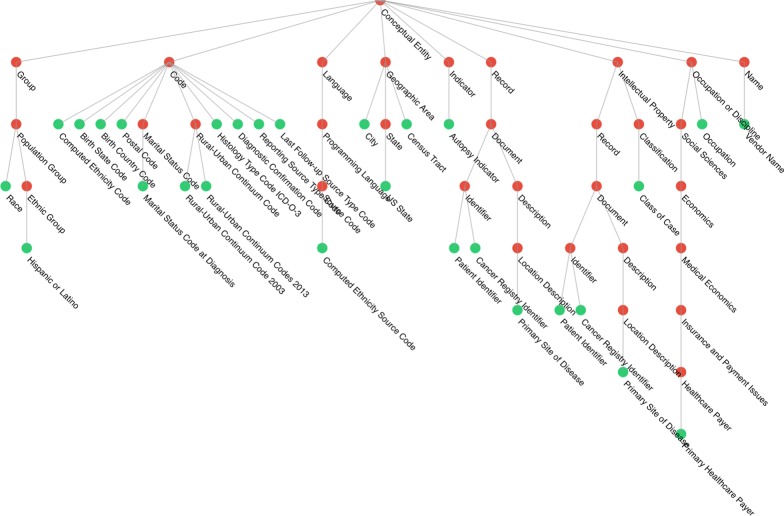
First branch of hierarchical structure

We identified five branches from NCIt for the extracted NAACCR data elements. Figure 4 shows an example of these branches, where green nodes denote concepts that have been mapped from the NAACCR data dictionary, and red nodes represent intermediate NCIt concepts. Table 2 summarizes the root concept, number of nodes, and maximum levels for these five branches. In IMI, we provide two modes for visualization. The first mode is a typical tree-based visualization. The second mode is the interactive mode with D3 library force layout. In the second mode, the root concepts are positioned in the central of the graph and users can interact with the graph by clicking and dragging nodes.

**Table 2:** Summary statistics of five branches

	B1	B2	B3	B4	B5
Root concept	Conceptual entity	Property or attribute	Disease, disorder or finding	Diagnostic or prognostic factor	Activity
No. of nodes	60	27	4	2	13
Maximum levels	7	5	3	1	8

### Mapping result review and exportation

**Fig. 5:**
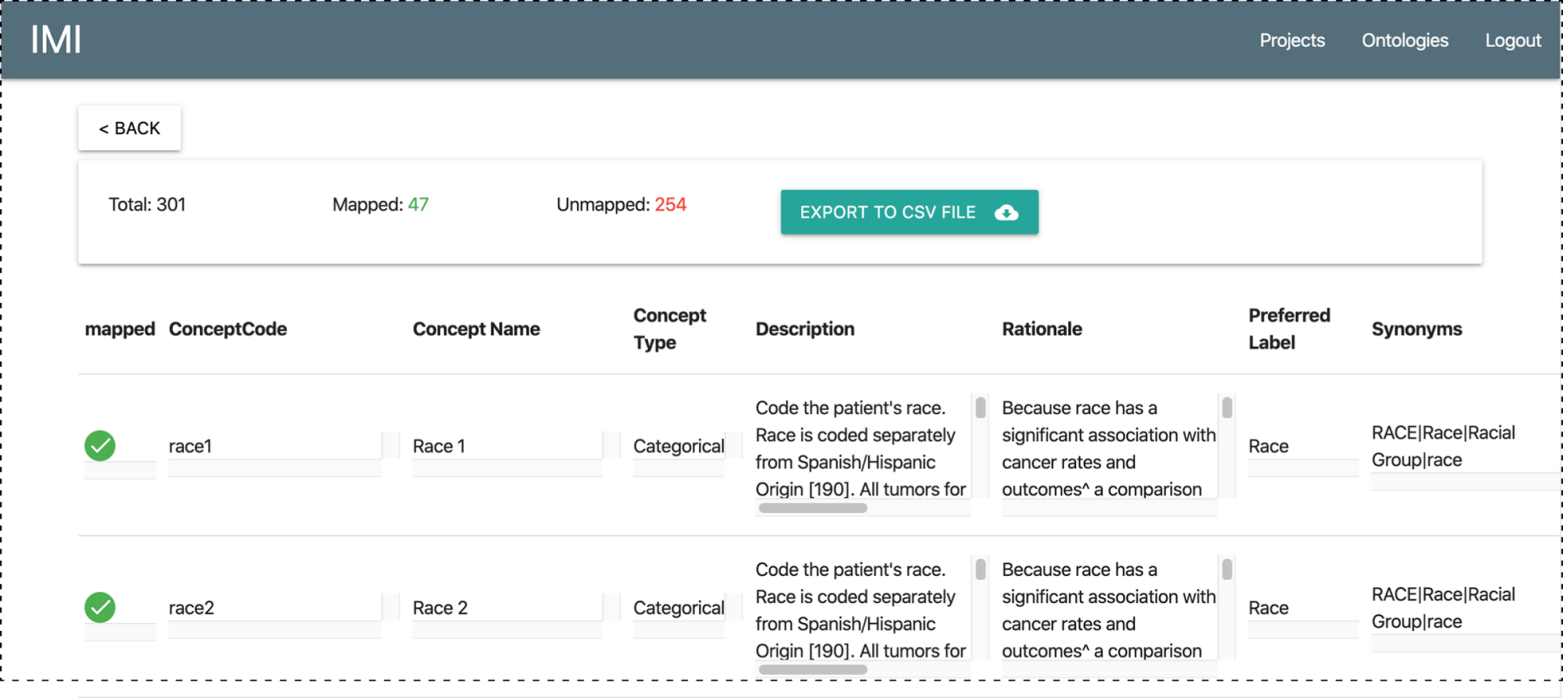
Mapping result review and export

The module for mapping result review and exportation summarizes the number of mapped and unmapped concepts as shown in Fig. 5. To export the mapping result, a user can simply click the “Export to CSV File” button to download the mappings as a CSV file.

### Comparative test of mapping efficiency

We selected ten variables from 301 variables in the NAACCR data dictionary, which were frequently used in KCR. We invited two independent researchers in the field of biomedical informatics but are not involved in the development of IMI. The two evaluators mapped the selected variables to NCIt using two approaches. The first approach utilized our IMI mapping interface and the second approach used the file-based mapping with spreadsheets. For the IMI-based approach, the evaluators selected each variable individually and searched possible matching concepts using our built-in searching function. For the file-based approach, the evaluators utilized the search function provided by the NCIt official website [20] to find potential matching concepts. Each evaluator performed the mappings for ten variables using the file-based approach and IMI-based approach, respectively. For each variable, the average mapping time taken (in seconds) by the two evaluators was calculated and reported. As shown in Table 3, the IMI-based approach is more efficient in terms of the time taken for performing the mappings. The two evaluators commented that during the mapping process they found the IMI interface was intuitive and user-friendly.

**Table 3:** Average mapping time for ten selected variables in the NAACCR data dictionary

NAACCR data dictionary variable	IMI-based approach (s)	File-based approach (s)	Mapped NCIt concept
Date of Birth	17.6	28.1	Birth Date
Race 1	12.3	30.6	Race
Sex	15.1	36.2	Sex
Race Coding Sys-Current	30.1	55.3	No mapping found
Race Coding Sys-Original	33.2	64.1	No mapping found
Spanish/Hispanic Origin	15.4	37.5	Hispanic or Latino
Birthplace-State	20.6	43.2	Birth State Code
Computed Ethnicity	17.1	29.7	Computed Ethnicity Code
Computed Ethnicity Source	18.1	40.3	Computed Ethnicity Source Code
Nhia Derived Hisp Origin	32.5	55.1	Hispanic or Latino

### Validity of the recommendation engine

To evaluate the validity of the recommendation engine, we compared the IMI’s automatic recommendation results with the mapping results verified by the KCR domain experts (authors IH, JM, and EBD) for the 47 NAACCR variables. When comparing the mapping results with the top candidate generated by the recommendation engine, 25 out of 47 (53%) recommendations were correct. Note that for certain data elements, the recommended mapping candidates actually had the same ranking score. Therefore, we also compared the mapping results with the top five recommended candidates, as a result of which 31 out of 47 (66%) recommendations were correct.

## Discussion

In our limited preliminary comparative study, we observed significant time–cost improvement of the IMI approach compared to a file-based approach. Since the NCIt website also provides a useful searching function, time for searching matching concepts did not make a significant difference. The difference rests in building mapping content. File-based approach requires additional time to enter search keywords, copy contents from the NCIt website, and paste them back to the spreadsheets, while IMI only requires a single click. We also observed that complete the mappings for certain concepts was more time-consuming when there were no corresponding matching concepts in the NCIt. Building mappings for such concepts requires additional validations. IMI provided features non-existent in the file-based approach, such as mapping result review and visualization.

### Limitations

In our experiment, only 47 (about 16%) of the NAACCR 301 variables were mapped to NCIt concepts. This low percentage is not necessarily a defect of IMI, as IMI only serves as an assistant to facilitate the mapping task. In fact, the semantic overlap between the source data dictionary and the target ontology represents a critical determinant of the mapping percentage. The low mapping percentage for our experiment may indicate that the NAACCR data dictionary and NCIt were designed for different needs, and many NAACCR variables have no corresponding concepts in NCIt.

Our comparative mapping study was limited to ten selected NAACCR data elements. Although among the most frequently used in KCR, they may not be representative for performance evaluation. Our purpose is to help highlight the differences of the two mapping approaches.

Our recommendation algorithm for matching data elements to ontology concepts is for demonstration of this possible feature. More sophisticated mapping algorithms may provide better recommendation results and further reduce the workload. For instance, we may further leverage embedding techniques in deep learning to match similar terms.

Currently, our IMI prototype only supports CSV file format for importing data dictionaries and ontologies. We plan to allow importing ontologies in the Web Ontology Language (OWL), a popular ontology representation format, in future work.

## Conclusions

In this paper, we presented IMI, an interactive mapping interface for building mappings from data dictionaries to ontologies, to facilitate semantic enrichment and support interoperability of metadata. IMI has been successfully pilot tested to construct mappings between the NAACCR data dictionary and NCIt. Although IMI’s interfaces were motivated for the KCR and cancer registries, its architecture has been designed to be generally applicable.

## Data Availability

The IMI system is available at https://epi-tome.com.

## Abbreviations

CSV: Comma-separated values
KCR: Kentucky Cancer Registry
NAACCR: North American Association of Central Cancer Registries
NCI: National Cancer Institute
NCIt: NCI Thesaurus
